# Exploiting ^19^F NMR in a Multiplexed Assay for Small GTPase Activity

**DOI:** 10.1021/jacs.4c14294

**Published:** 2024-12-18

**Authors:** Fatema Bhinderwala, Angela M. Gronenborn

**Affiliations:** Department of Structural Biology, University of Pittsburgh School of Medicine, Pittsburgh, Pennsylvania 15260, United States

## Abstract

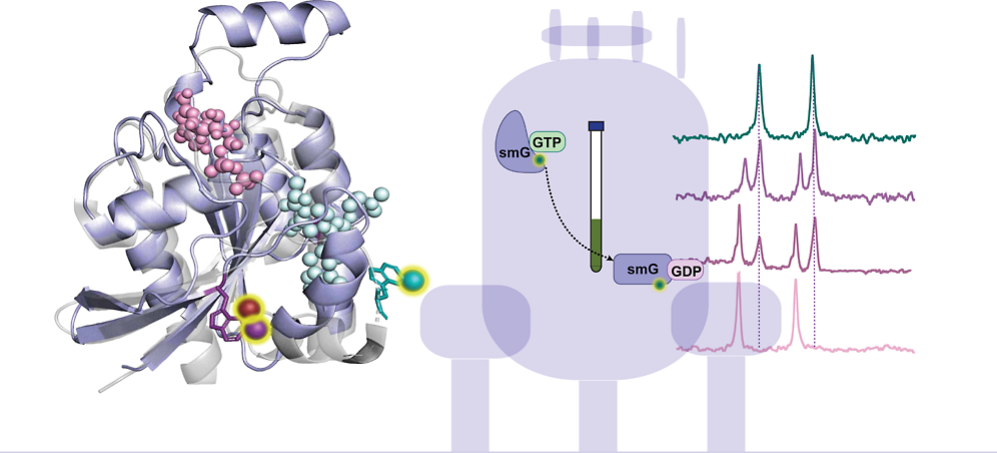

Small GTPases (smG) are a 150-member family of proteins, comprising five subfamilies: Ras, Rho, Arf, Rab, and Ran-GTPases. These proteins function as molecular switches, toggling between two distinct nucleotide-bound states. Using traditional multidimensional heteronuclear NMR, even for single smGs, numerous experiments, high protein concentrations, expensive isotope labeling, and long analysis times are necessary. ^19^F NMR of fluorinated proteins or ligands can overcome these drawbacks. Using indole position-specific ^19^F labeling of the proteins, the activities of several smGs were measured in a multiplexed fashion. We investigated 4-, 5-, 6-, and 7-fluoro tryptophan containing smGs to study nucleotide binding. Distinct resonances for GDP- or GTP-bound states of three different ^19^F-labeled smGs, RhoA, K-Ras, and Rac1, were observed, and the kinetics of exchange and hydrolysis were measured. This multiplexed system will permit screening of nucleotide-specific ligands of smGs under true physiological conditions.

## Introduction

Small GTPases (smG) are molecular switches that interconvert between GDP-bound and GTP-bound states, regulating a variety of interconnected signaling cascades.^1,2^ The intrinsically slow rate of GTP hydrolysis and GDP to GTP exchange in smG proteins is accelerated by activating proteins (GAP)^3^ and guanine nucleotide exchange factors (GEFs).^4^ Nucleotide exchange is accompanied by a conformational change in the switch II region and the effector loop, resulting in GDP and inorganic phosphate release, which resets the protein for reloading of the GTP substrate to complete the cycle.^5,6^ K-Ras regulates cell growth and proliferation via the PI3K pathway, and RhoA controls cytoskeleton remodeling^7,8^ through actin fibrillation, aiding overall cell survival.^7^ Rho GTPases are involved in tumor progression and angiogenesis in breast, melanomas, gastric, and liver cancers.^9,10^ Similarly, aberrant Rac1 interactions with its GEFs, like Tiam and ARFGEF, cause tumorigenesis.^8,11,12^ One smG can activate another smG through shared regulatory processes in cell signaling pathways.^13^ In essence, smG proteins function synchronously and are regulated by a fine-tuned interplay to safeguard cellular homeostasis and health.^4^ Unfortunately, most studies evaluating smG’s activities and potential drug-binding capabilities, are carried out with isolated components,^14^ missing important context-dependent effects. Existing methods for measuring nucleotide exchange and GTP hydrolysis of individual smG proteins rely on either fluorescence-, calorimetry- or radioactive substrate-based assays, each of which has some drawbacks. For example, fluorescence-based methods employ fluorescently tagged substrate analogs or proteins, with readouts being influenced by the nature and location of the fluorescent probes,^15^ and, in the case of BODIPY-tagged GTPγS or GDP, nonspecific interactions with buffer components are known to impact hydrolysis and exchange kinetics.^16^ In addition, most of the time, only a single smG can be monitored. Given the above limitations, complementary alternative approaches are highly desirable.

NMR provides such an alternative. Using uniform ^15^N and/or ^13^C labeling, extensive backbone and side chain assignments are necessary to identify reporter resonances for GTP loading or hydrolysis.^17,18^ Frequently, specific amino acids in smG proteins are isotopically labeled, alleviating spectral overlap.

Most of the inherent limitations of existing ^15^N and/or ^13^C NMR-based GTPase activity assays are overcome by using protein-detect ^19^F NMR experiments. ^19^F is an excellent reporter nucleus for NMR studies due to its high sensitivity (83% of ^1^H) and large chemical shift range (∼300 ppm).^19^ Also, the ^19^F chemical shift is exquisitely sensitive to the local environment, making it ideally suited to report on ligand or protein binding-induced conformational changes and/or motional differences.^20−22^ From a practical standpoint, one-dimensional (1D) ^19^F NMR spectra are sufficient, fast, adaptable, and robust since resonance overlap is less of an issue with only a few resonances, resulting in significant time savings if real-time monitoring of reactions is desired. Here, we present a real-time ^19^F NMR assay that uses fluorotryptophans to report on nucleotide exchange and GTP hydrolysis.

## Results and Discussion

### ^19^F Trp Probes are Nucleotide State Sensitive and Report on GTP Hydrolysis in smG Proteins

**Figure 1:**
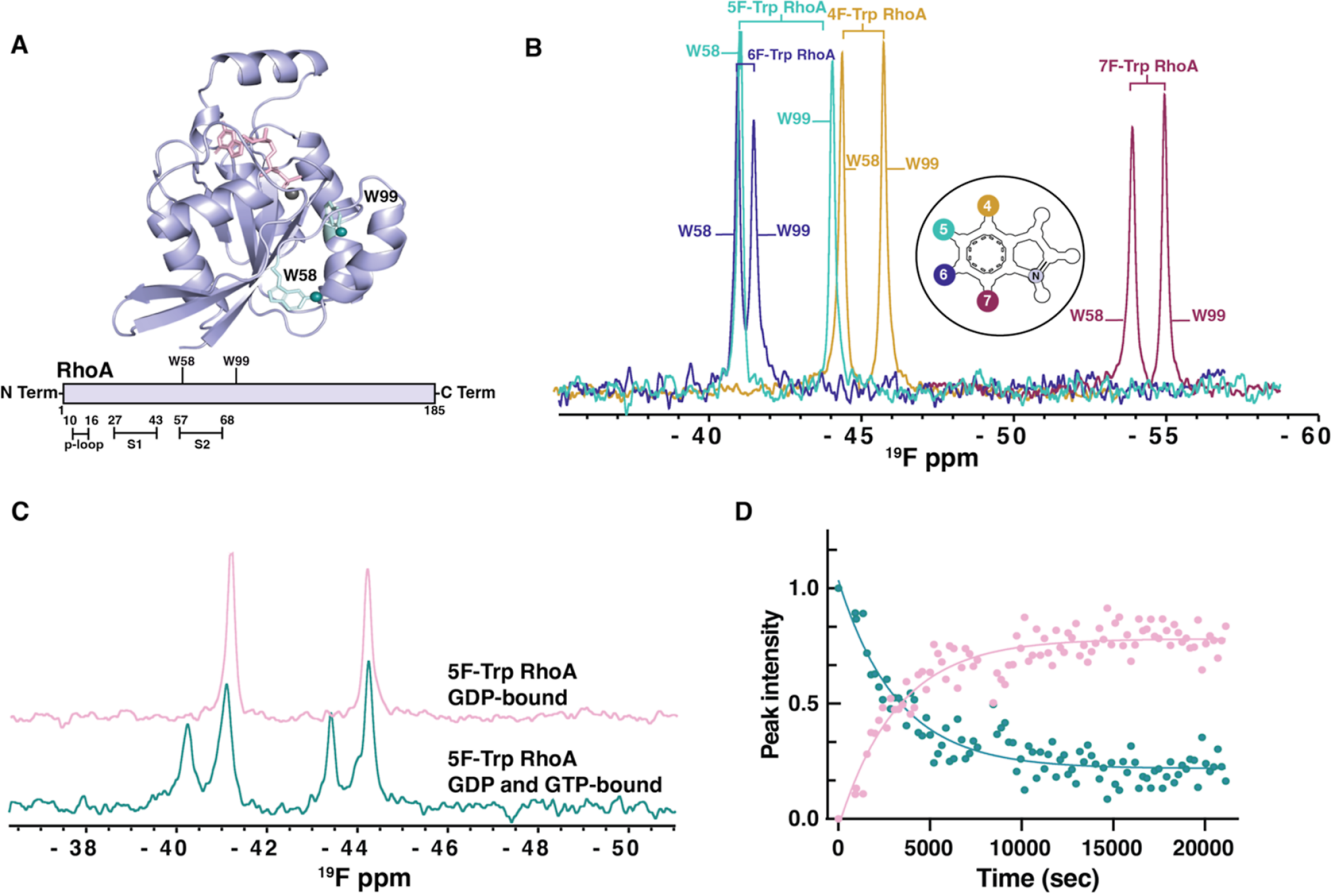
(A) Top: ribbon diagram of the RhoA protein structure (PDB: 1FTN) bottom: schematic depiction of RhoA organization. (B) Superposition of the 1D ^19^F spectra for four differently labeled F-Trp containing RhoA proteins (4F, yellow; 5F, teal; 6F, purple; 7F, magenta). The chemical structure of the Trp indole ring (inset circle), with positions 4, 5, 6, and 7 labeled in yellow, cyan, purple, and magenta. (C) 1D ^19^F spectra of GDP-bound 5F-TrpRhoA protein (pink) and a mixture of GDP- and GTP-bound 5F-TrpRhoA (green). (D) Time dependence of peak intensities (sum of Trp58 and Trp99 resonances) of the GTP-bound (green) and GDP-bound (pink) 5F-TrpRhoA protein.

Three smG proteins were fluorinated on the tryptophan indole ring by amino acid type-specific labeling. Tryptophan residues are inherently sparse in proteins with the lowest overall amino acid abundance in the human proteome, limiting any possible adverse effects of introducing too many fluorines.^23^ Adding 4-, 5-, 6-, or 7-fluoroindole to the growth medium permits routine production of differentially labeled fluorotryptophan (F-Trp) proteins in *Escherichia coli*.^24^ Another advantage of using F-Trp as a reporter is that incorporation of 4-, 5-, 6-, or 7-fluoro tryptophan is minimally perturbing in systems ranging from small well-folded proteins to larger transmembrane receptors.^25,26^ We first explored the feasibility of using ^19^F-labeled smG proteins for monitoring nucleotide exchange and hydrolysis using the Ras homology protein A (RhoA). RhoA possesses two tryptophans, W58 and W99, both positioned within 20 Å of the nucleotide-binding pocket, flanking the switch II region that undergoes a large conformational change upon nucleotide exchange (Figure 1A). We prepared ∼100% fluorine labeled 4-, 5-, 6-, and 7F-TrpRhoA, as confirmed by mass spectrometry (Figure S1). The ^1^H, ^15^N HSQC spectra of the fluorinated proteins reveal minimal changes in chemical shifts, confined to the amide resonances of amino acids that surround the Trp substitution (shown for RhoA in Figure S2), indicating no significant change in the overall structures. The ^19^F spectra of all four F-Trp labeled RhoA proteins exhibit two resonances with comparable intensities and line widths, ranging from 80 to 140 Hz (Figure 1B). 5F-TrpRhoA exhibits the largest difference in ^19^F resonance frequencies (∼1 ppm) for the two tryptophan residues, compared to 4-, 6-, and 7F-TrpRhoA (Figure 1B). Resonance assignments for 5F-TrpRhoA were readily obtained by mutagenesis, replacing each Trp by Phe (Figure S3). To assess whether the fluorinated residues report on the nature of the bound nucleotide, GDP was exchanged to GTP in each of the F-TrpRhoA samples, and characteristic downfield shifts were observed for both ^19^F resonances in all spectra (Figure 1C). For 5F-TrpRhoA, the GDP bound signals for W58 and W99 move from −40.95 and −44.05 ppm to −40.20 and −43.40 ppm, respectively (Figure 1C). By contrast, 4F-TrpRhoA experiences little chemical shift changes upon GTP binding and, in this case, one of the resonances shifts downfield from −45.64 to −44.20 ppm, while the other shifts upfield from −44.40 to −44.95 ppm (Figure S4A). For 6F-TrpRhoA, both W58 and W99 resonances shift downfield from −42.51 and −41.95 ppm to −41.45 and −40.91 ppm upon GTP binding (Figure S4B). Lastly, for 7F-TrpRhoA, the W58 resonance at −54.9 ppm is insensitive to GTP binding, while the W99 resonance broadens and moves downfield from −53.88 to −51.47 ppm (Figure S4C). The intrinsic hydrolysis of GTP-bound RhoA was followed in a series of real-time 1D ^19^F experiments for 5F-TrpRhoA, monitoring peak intensity changes from GTP- to GDP-bound RhoA as a function of time (Figure 1D; 7F-TrpRhoA data are shown in Figure S5). Similarly, intrinsic nucleotide exchange from GDP to GTPγS was followed for 4F-TrpRhoA and 5F-TrpRhoA (Figure S6). These experiments established the feasibility of using F-Trp probes in RhoA to report on both nucleotide exchange (Figure S7) as well as GTP hydrolysis by NMR.

**Figure 2:**
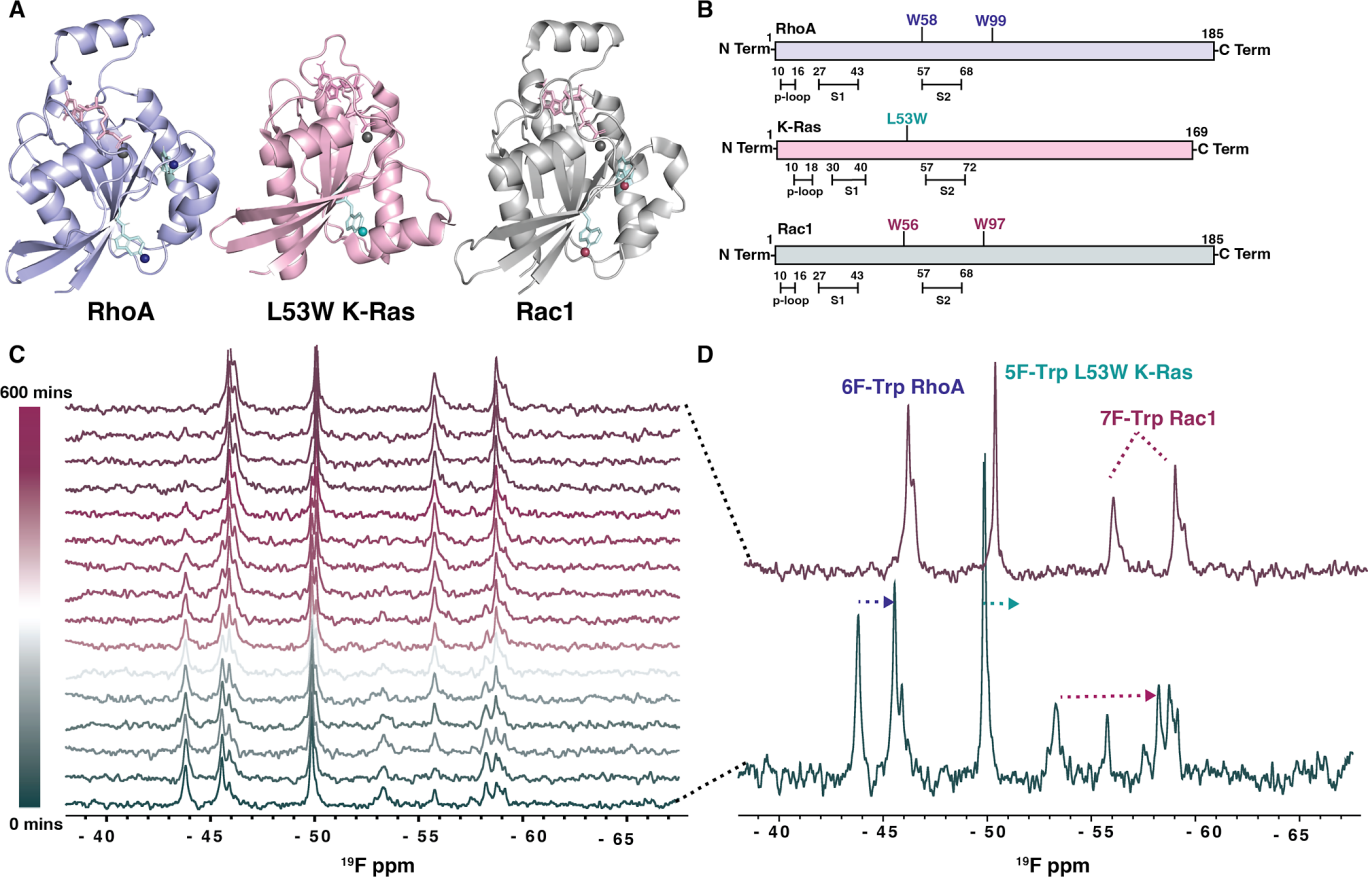
(A) Ribbon diagrams of the K-Ras, RhoA, and Rac1 protein structures (PDB ID: 4OBE, 1FTN, 5N6O) with Trp side chains as sticks and fluorine atoms as spheres. Bound GDP is colored pink. (B) Schematic depiction of the organization of all three proteins (C) time-dependent series of 1D ^19^F spectra (green to magenta) of a mixture of the three small GTPase proteins (5F-Trp L53WK-Ras, 6F-TrpRhoA, and 7F-TrpRac1) following GTP hydrolysis. (D) 1D ^19^F spectra of the mixture of smG proteins at the beginning (GTP- and GDP-bound; green) and end (GDP-bound magenta) of the time-dependent series.

To test whether our approach holds for other smG proteins, we selected Rac1, another Rho GTPase. Rac1 also possesses two conserved Trp residues at positions 56 and 97 (Figure 2). 1D ^19^F spectra of 6F-Trp and 7F-TrpRac1 show two resolved resonances at −42.0 and −41.6 ppm (6F-TrpRac1) or at −56.2 and −53.8 ppm (7F-TrpRac1) (Figures S8, S9A, S9B). Both 7F-TrpRac1 and RhoA exhibit similar ^19^F spectra upon GTP binding (Figures S4C and S9B), with one resonance essentially unaffected and the other broadening and shifting downfield from −53.8 to −50.65 ppm. Thus, these F-Trp labeled smG proteins are suitable for ^19^F NMR-based monitoring of nucleotide cycling (Figure S10).

### Development of a Multiplexed Hydrolysis Assay for smG using ^19^F Trp Probes

In the cellular context, several smG Proteins are present at the same time. Therefore, we sought to establish that GTP hydrolysis can be followed for a mixture of smG proteins. Unfortunately, K-Ras and other members of the Ras protein family do not possess any Trp residues. It therefore was necessary to introduce a single Trp residue into a site structurally equivalent to W58 in RhoA. Based on the crystal structures of RhoA, Rac1, and K-Ras (Figure 2A), we introduced a L53W change into K-Ras (Figure 2B). This variant was previously shown not to adversely affect K-Ras activity.^27^ 5F-TrpL53W K-Ras exhibits a W53 resonance at −43.21 ppm in the GDP-bound state, and this resonance moves downfield to −42.83 ppm upon GTP binding (Figure S9C). Therefore, 5F-TrpL53W K-Ras can be used to follow GTP hydrolysis by ^19^F NMR (Figure S10C). Having three differently fluorinated smG proteins at hand allowed us to measure GTP hydrolysis in a mixture of these proteins in a multiplexed assay. To minimize resonance overlap, we used 5F-TrpL53W K-Ras, 6F-TrpRhoA, and 7F-TrpRac1 to follow the proteins from GTP- to GDP-bound states. Gratifyingly, the proteins in the mixture behave essentially the same as in isolation, as evidenced by similar line widths, chemical shifts, and rate of hydrolysis (Figures 2C, S10B–D and S16), rendering ^19^F NMR ideally suited to carry out experiments in a multiplexed fashion, unimpeded by possible resonance overlap. This is particularly important when considering the possibility of assaying proteins in native or near-native conditions. For example, using traditional ^15^N, ^13^C NMR methods, such assays would be impossible in cell lysates with protein concentrations close to native, given all the components in cell lysates.

In fact, traditional 2D ^1^H–^15^N or ^1^H–^13^C HSQC spectra contain only a handful of resonances for smG proteins when crowding agents are present (Figure S11A), while all resonances in 1D ^19^F spectra are easily observed (Figure S11B,C) and largely unchanged under such conditions. Owing to the high solubility of small GTPases, these experiments are readily carried out at micromolar concentrations (30–100 μM) in very short times, yielding a time resolution of a few minutes for the time-dependent measurements. This may not always be possible for proteins that are poorly soluble. In such cases, the temporal resolution will be limited by the overall protein concentrations that can be achieved in single and multiplexed NMR experiments.

### Advantages of ^19^F NMR to Evaluate Drug Binding to Oncogenic K-Ras

**Figure 3:**
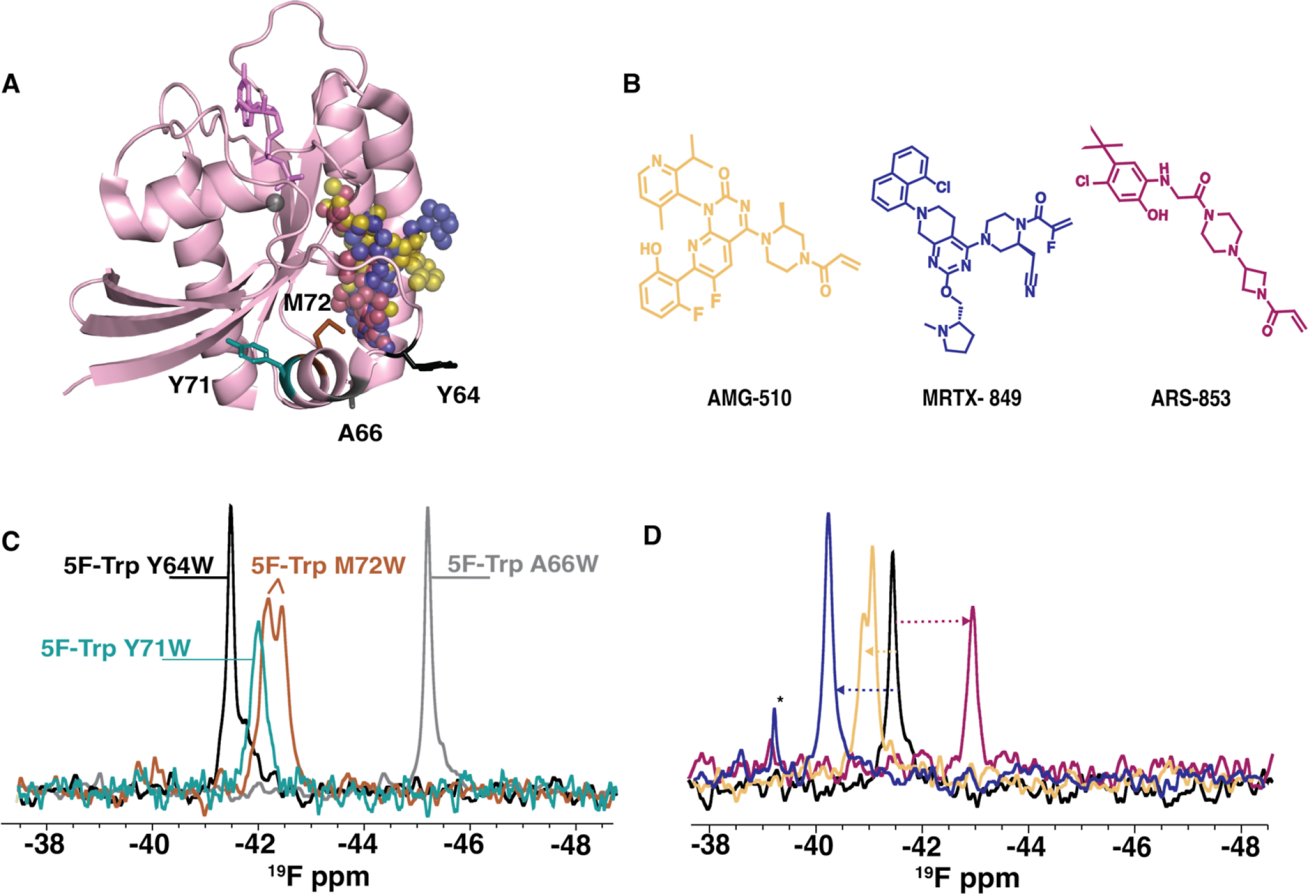
(A) Ribbon diagram of the G12C K-Ras crystal structures with AMG-510 (yellow), MRTX-849 (cyan), and ARS-853 (magenta) inhibitors (PDB: 6OIM, 6UTO, and 6USZ). Amino acids that were changed to Trp are labeled. (B) Chemical structures AMG-510 (yellow), MRTX-849 (purple), and ARS-853 (magenta). (C) Superposition of 1D ^19^F spectra of 5F-Trp G12C K-Ras variants. (D) Superposition of 1D ^19^F spectra of 5F-Trp Y64W, G12C K-Ras (black) with AMG-510 (yellow), MRTX-849 (purple), and ARS 853 (magenta). *, free fluoride.

Previously, diverse and innovative strategies have been pursued for developing K-Ras inhibitors, such as targeting nucleotide exchange, binding and stabilizing nonproductive Ras complexes, and decreasing Ras-effector binding.^28^ The difficulties in targeting K-Ras are predominantly associated with the lack of drug-binding pockets around switch II.^29^ The first specific drug for G12C K-Ras to treat nonsmall cell lung cancer was FDA-approved only in May 2021.^30^ Several other inhibitors, such as MRTX-849, JNJ-7469915721, and GDC-6039, are at different stages in preclinical development and clinical trials.^31−33^ The success of these inhibitors lies in their covalent attachment to the cysteine residue in the GDP-bound G12C K-Ras.^34^ The structures of K-Ras bound to such covalent inhibitors shows the ligands near the switch II region in a cryptic pocket (Figure 3A).^28,35^ AMG-510 ARS-853 and MRTX-849 (Figure 3B) are selective for the local structure around C12 in G12C K-Ras, but not in wild-type K-Ras. Based on the available structural details,^36^ we introduced a new Trp residue close to the cryptic binding pocket in G12C K-Ras (Figure 3A). Y64, A66, Y71, and M72 were tested as possible positions for substitution, and four F-Trp variants were prepared (Figure S11). The A66W and M72W G12C K-Ras variants exhibited significantly broader resonances (∼250 Hz) than 5F-TrpY64W G12C K-Ras (∼90 Hz) (Figure 3C). Thus, Y64W was selected for further studies.

We tested AMG-510, MRTX-849 and ARS-853 for their effects on the ^19^F spectrum of 5F-TrpY64W G12C K-Ras (Figures 3D and S13). As can be appreciated, each inhibitor imparts a unique signature onto the spectrum: AMG-510 binding results in a 1.2 ppm downfield shift, while MRTX-849 induces a 0.45 ppm downfield shift, and ARS-853 causes a 1.45 ppm upfield shift (Figure 3D). Importantly, 5F-TrpY64W G12C K-Ras is sensitive to nucleotide binding (Figure S14A), with the 5F-Trp64 resonance shifting downfield from −44.20 ppm in GDP-bound state to −43.56 ppm in GTP-bound state. Therefore, 5F-TrpY64W K-Ras can be used in assays to evaluate GTP hydrolysis (Figure S14B). We also tested AMG-510 and ARS-853 binding to oncogenic 5F-TrpL53W G12C K-Ras. However, no significant chemical shift changes occurred (Figure S15), rendering this variant not a viable candidate for drug binding studies, in contrast to 5F-TrpY64W G12C K-Ras.

In summary, we present a powerful ^19^F NMR-based real-time NMR approach for simultaneously measuring nucleotide exchange, hydrolysis, and ligand binding in smG proteins. Exploiting the exquisite sensitivity of ^19^F NMR uniquely permitted us to measure GTP hydrolysis in a multiplexed fashion, which is difficult using fluorescence-based methods. In addition, although we showcase the power of the ^19^F NMR-based real-time NMR approach for screening only a few selected smG proteins, the general methodology is easily transferable to other enzymes, such as ATPases and kinases. Our investigation is a first-of-its-kind enzyme function study, taking advantage of the unique chemical shift ranges for 4F-, 5F- and 6F-Trp resonances. It adds another tool to the growing repertoire of NMR approaches used for fragment-based drug discovery, like RAMPED-UP NMR^37^ and ProF-NMR.^38^ Altogether, these methodologies will undoubtedly inspire further applications where incorporating fluorinated amino acids does not alter the properties of the enzyme system at hand. We anticipate rapid developments, especially with expanding protein ^19^F labeling strategies and redeploying such assays into physiologically relevant cancer cells.

## Supporting Information Available

Material and Methods, NMR data acquisition and processing procedures, ^19^F and 2D ^1^H–^15^N NMR spectra, and LC–MS data (PDF)
