# Supplementary material for: Exploiting 19F NMR in a Multiplexed Assay for Small GTPase Activity
Source: J Am Chem Soc. 2024 Dec 18;147(1):1028–33. doi: 10.1021/jacs.4c14294 (PMC11719398; doi:10.1021/jacs.4c14294)
Supplement: Supplementary file 1 — ja4c14294_si_001.pdf [file ja4c14294_si_001.pdf]

Exploiting ^{19}F NMR in a multiplexed assay for small GTPase activity

Fatema Bhinderwala,[1] Angela M. Gronenborn[1*]

[1] Department of Structural Biology, University of Pittsburgh School of Medicine, Pittsburgh, PA 15213, United States

[*]To whom correspondence should be addressed:

Angela M. Gronenborn
UPMC Rosalind Franklin Professor,
Department of Structural Biology,
University of Pittsburgh School of Medicine,
amg100@pitt.edu

Table of Contents

Materials and Methods

Figure S1: Mass spectrometry data for RhoA

Figure S2: 2D ^{1}H-^{15}N HSQC spectra of RhoA

Figure S3: Mass spectrometry data and 1D ^{19}F spectra for Trp-Phe substituted RhoA

Figure S4: 1D ^{19}F spectra for GDP-bound and GTP-bound RhoA

Figure S5: Time-dependent 1D ^{19}F spectra of 7F-Trp RhoA following GTP hydrolysis

Figure S6: Time-dependent 1D ^{19}F spectra of 4F-Trp RhoA and 5F-Trp RhoA following nucleotide exchange

Figure S7: Nucleotide exchange assay for RhoA and 5F-Trp RhoA using fluorescence and ^{19}F NMR

Figure S8: Mass spectrometry data for Rac1 and K-Ras

Figure S9: 1D ^{19}F spectra of GDP-bound and GTP-bound Rac1 and K-Ras

Figure S10: Time-dependent 1D ^{19}F spectra of 7F-Trp RhoA, 7F-Trp Rac1 and 5F-TrpL53W K-Ras following GTP hydrolysis

Figure S11: 2D ^{1}H-^{15}N HSQC and 1D ^{19}F spectra of RhoA and K-Ras in crowded environments

Figure S12: Mass spectrometry data for Trp variants of K-Ras

Figure S13: Mass spectrometry data for ligand conjugated 5F-TrpY64W G12C K-Ras

Figure S14: 1D ^{19}F spectra of GDP-bound and GTP-bound 5F-TrpY64W K-Ras and corresponding time-dependent GTP hydrolysis

Figure S15: 1D ^{19}F spectra of 5F-Trp L53W G12C K-Ras in the presence of the inhibitors AMG-510 and ARS-853.

Figure S16: GTPase activity of 6F-Trp RhoA measured alone and in the multiplexed assay

Materials and Methods

Materials All chemicals were purchased from MilliporeSigma (Sigma-Aldrich, St. Louis, MO, USA), unless otherwise stated. D_2O and $^{15}NH_4Cl$ were purchased from Cambridge Isotopes (Tewksbury, MA, USA). Guanosine nucleotides GDP and GTP were purchased from Life Technologies (Vilnius, Lithuania), BODIPY-FL-GDP from Invitrogen (Eugene, OR, USA) and GTPγS from Sigma-Aldrich (St. Louis, MO, USA). All K-Ras inhibitors were purchased from MedChemExpress (Monmouth Junction, NJ, USA).

Plasmids and expression constructs

The RhoA (amino acids (aa) 1-181) - encoding insert in pNIC28-Bsa4 and Rac1(aa 1-180) -encoding insert in pNIC-NStIIT were purchased from Addgene (Watertown, MA). A DNA insert encoding K-Ras4b (aa 1-169), containing an N-terminal 6x His tag followed by a TEV protease cleavage site, was purchased from Genscript (Piscataway, NJ, USA) and inserted into pet28b (+) using restriction sites Xho1 and Nde1. All other constructs were created by site-directed mutagenesis using overlapping primers.

Site-directed mutagenesis for specific K-Ras and RhoA mutants

The G12C K-Ras4b (aa 1-169) tryptophan variants (L53W, Y64W, A66W, Y71W, and M72W), along with W58F RhoA and W99F RhoA were obtained by a site-directed mutagenesis approach using the polymerase chain reaction with PFII Ultra (Agilent Technologies, Santa Clara, CA, USA) and forward and reverse primers designed to cover the mutagenesis sites. Primers with the desired nucleotide changes flanked by five codons on either side were purchased from Integrated DNA Technologies (Coralville, IA, USA). All mutants were confirmed by sequencing before use.

Protein expression and purification for N-terminal 6x His-tagged proteins RhoA, K-Ras and Rac1

^{19}F-Trp,^{15}N RhoA, Ras, Rac1 proteins and their variants were expressed in *E. coli* BL21 (DE3) (New England Biolabs, Ipswich, MA, USA), cultured in modified M9 medium, containing 4 g/L U-$^{12}C_6$-glucose, 1 g/L $^{15}NH_4Cl$, and 20 mg/L 4-, 5-, 6-, or 7-fluoroindole as carbon, nitrogen, and fluorine sources. Cultures were grown to ~ 0.7- 0.8 A_{600} and induced with 0.75 mM IPTG for protein expression at 18 °C for 16 h. The labeling method was similar to that previously described.[1, 2] In essence, cells were harvested by centrifugation at 4000×g for 25 min at 4 °C, resuspended in 25 mM sodium phosphate buffer with 250 mM sodium chloride (NaCl), 5 mM magnesium chloride ($MgCl_2$), and 35 mM imidazole (pH 8.0), and ruptured by sonication. Cell debris was removed by centrifugation at 27,000×*g* for 1 hour at 4 °C. The pH of the supernatant was checked and adjusted with NaOH to pH 8.0, when necessary. Following clarification of the lysate, the final supernatant was loaded onto a Ni His-affinity column

(HiTrap HisTrap FF, 5 mL, GE Healthcare, Chicago, IL, USA). After an initial wash with 60 mM imidazole, His-tagged proteins were eluted using 1M imidazole in a buffer containing 25 mM sodium phosphate (pH 8.0), 1 mM DTT, and 0.02% NaN_3. Concentrated protein fractions were further purified using a size-exclusion column (HiLoad 26/600 Superdex 75, GE Healthcare,Chicago, IL, USA), equilibrated in 25 mM sodium phosphate buffer (pH 6.5), 150 mM NaCl, 5 mM $MgCl_2$, 1 mM DTT, 0.02% NaN_3. At this stage, proteins were treated with Tobacco Etch Virus protease (TEVP) at 1:10 molar ratio of TEVP:protein, to cleave off the N-terminal His_6-affinity tag. The cleaved protein was purified further over a 5 ml HiTrap HisTrap FFcolumn (GE Healthcare, Chicago, IL, USA) and the flow-through was collected. Following this step, the protein samples were concentrated using an Amicon concentrator and buffer exchanged into 25 mM HEPES, pH 7.0, 150 mM NaCl, 5 mM $MgCl_2$, 0.5 mM TCEP (HEPES A).

Protein purification for N-terminal Strep-tagged Rac1

The Strep-tagged Rac1 was expressed as above, and cells were lysed in buffer containing 25 mM Tris buffer, 150 mM NaCl, and 5 mM $MgCl_2$. After clarification by centrifugation, the supernatant was loaded onto a Strep affinity column (HiTrap Strep HP column, GE Healthcare, Chicago, IL, USA) and eluted with 125 mM desthiobiotin. The protein was further purified over a Superdex 75 column (GE Healthcare, Chicago, IL, USA) in 25 mM sodium phosphate buffer, pH 7.0, 150 mM NaCl, 5 mM $MgCl_{2,}$ 1 mM DTT, 0.02% NaN_3 for TEV cleavage. The protein was treated with Tobacco Etch Virus protease (TEVP) at 1:10 molar ratio of TEVP:protein, to cleave off the N-terminal Strep-affinity tag. The cleaved protein was purified further over a 5ml HiTrap HisTrap FFcolumn (GE Healthcare, Chicago, IL, USA) and the flow-through was collected. Following this step, the protein samples were concentrated using an Amicon concentrator and buffer exchanged into HEPES A.

Nucleotide exchange assay using BODIPY-tagged fluorescent GDP

100 µM purified GDP-bound RhoA protein in 20 mM HEPES buffer, 150 mM NaCl, 6 mM EDTA, pH 7.0 was incubated with 500 µM of BODIPY FL-GDP at 37 °C for ~ 45 minutes for exchanging the native nucleotide to the fluorescent BODIPY-FL GDP, followed by addition of 10 mM $MgCl_2$ to quench EDTA. Excess fluorescent nucleotide was removed by 30-fold dilution of the sample into 25 mM HEPES, 150 mM NaCl, 5 mM Mg Cl_2, pH7.0. The sample was flash frozen until used in the exchange assay. For the assay, 50 µL of 1 µM RhoA was incubated with 10 µM GTP-γS (10-fold excess) in the presence of 5 mM $MgCl_{2.}$ The change in fluorescence intensity was monitored in a 96-well plate at 1-minute intervals for 180 minutes using a TECAN Spark™ multiwavelength plate reader (TECAN, Männedorf, Switzerland) using an excitation wavelength of 500 nm and an emission wavelength of 515 nm (± 5 nm). For data

processing, the fluorescence intensity of free 2 μM BODIPY FL-GDP in HEPES buffer was used as the original signal. Fluorescent intensities were normalized as percentages of free 2 μM BODIPY FL-GDP. Nonlinear regression fitting to an exponential decay model, using Prism 10 (GraphPad Software), was applied to obtain the pseudo-first-order rate constant.

Nucleotide exchange assay for NMR experiments

Proteins were incubated at 37 °C with 6 mM EDTA-Na and a 10-fold excess of the desired nucleotide for 45 minutes. Subsequently, the sample was placed on ice, and 15 mL of HEPES A buffer was added. The resulting protein sample was then concentrated in an Amicon concentrator to the required protein concentration and used immediately to record spectra to follow nucleotide exchange.

Conjugation of inhibitors to K-Ras Trp variants

Proteins were incubated at 25 °C for 45 minutes with 5 molar equivalents of inhibitor, dissolved in DMSO. Thereafter, the sample was placed on ice and the inhibitor-protein conjugate samples were used immediately to record NMR spectra. A portion was diluted in HEPES A buffer for mass spectrometry analysis.

NMR data acquisition and processing

^{19}F experiments

All samples for ^{19}F NMR were prepared at 100 μM protein concentration. ^{19}F NMR spectra were recorded on a 14.1 T Bruker AVANCE spectrometer equipped with a TXO F/C–H–D triple-resonance, z-axis gradient cryoprobe at 298 K. The temperature was maintained using the Bruker temperature control unit. For single protein GTP hydrolysis experiments, 1D ^{19}F spectra were measured at 100 μM protein concentration using a spectral width of 28409 Hz with 16,000 points, 256 scans and a recycle delay of 1 sec. All spectra were processed using Topspin 4.1 (Bruker). For the multiplexed experiments, 1D ^{19}F spectra were measured at 33 μM protein concentration for each of the three proteins (100 μM total protein concentration) using a spectral width of 28409 Hz, 16,000 points, 512 scans and a recycle delay of 1 sec. Time-dependent series of spectra were processed using an exponential window function and a line broadening of 100 Hz. The data were phased and baseline corrected, and peak intensities were fit using MNova (MestreLabs, San Diego, CA, USA). Data were plotted and fit to a single exponential decay function by Prism 10 (GraphPad Software, La Jolla, CA, USA)

2D ^{1}H-^{15}N HSQC spectra

^{1}H-^{15}N HSQC spectra were recorded on a 14.1 T Bruker AVANCE spectrometer equipped with a TCI HCN triple resonance cryoprobe. All samples were prepared similar to those used for ^{19}F NMR. 2D ^{1}H-^{15}N HSQC spectra were measured with a ^{1}H spectral width of 9645 Hz and ^{15}N spectral width of 3245Hz, using a recycle delay of 1 sec. All spectra were processed using Topspin 4.1 (Bruker).

Mass Spectrometry data acquisition and processing

ESI LC-MS measurements were performed at 1 μM protein concentration on a Bruker Q-TOF instrument, using a reverse phase AdvanceBio peptide guard column (Agilent Technology) where mobile phases A and B comprised 5% acetonitrile and methanol with 0.01% Formic Acid, respectively. The resulting LC-MS spectra were processed using Bruker Compass Software, and the MS data were processed using maximum-entropy-based deconvolution to obtain the M+ ion mass for each sample. The instrument was calibrated using the ESI Low Tuning mix I (Agilent Technologies) to 1.0 ppm mass % difference before each use.

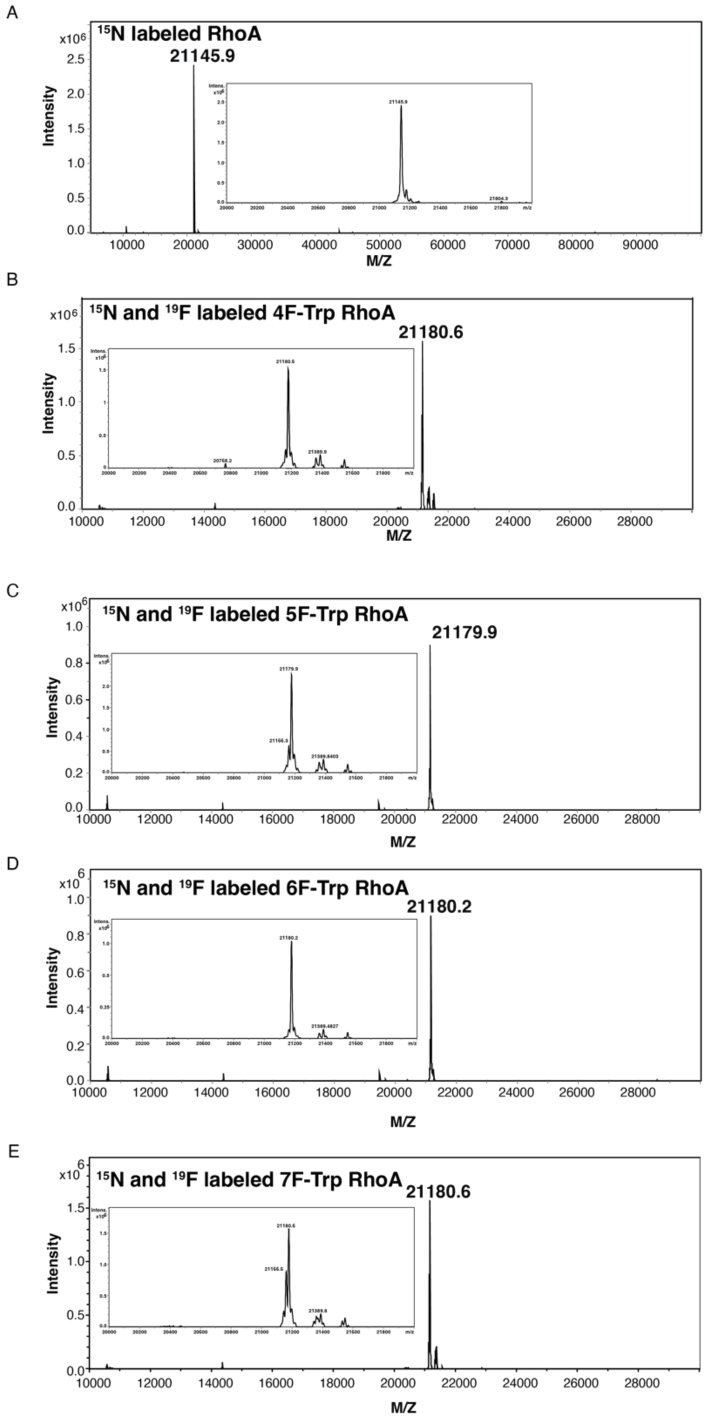

Figure S1: ESI Mass spectrometry data for (A) ^{15}N RhoA-WT and (B, C, D, and E) ^{15}N, ^{19}F labeled RhoA (4-,5-,6-,7-F Trp). The calculated expected masses are 21145 Da and 21182 Da, respectively. Insets show the deconvoluted mass spectra between 20000-22000 Da.

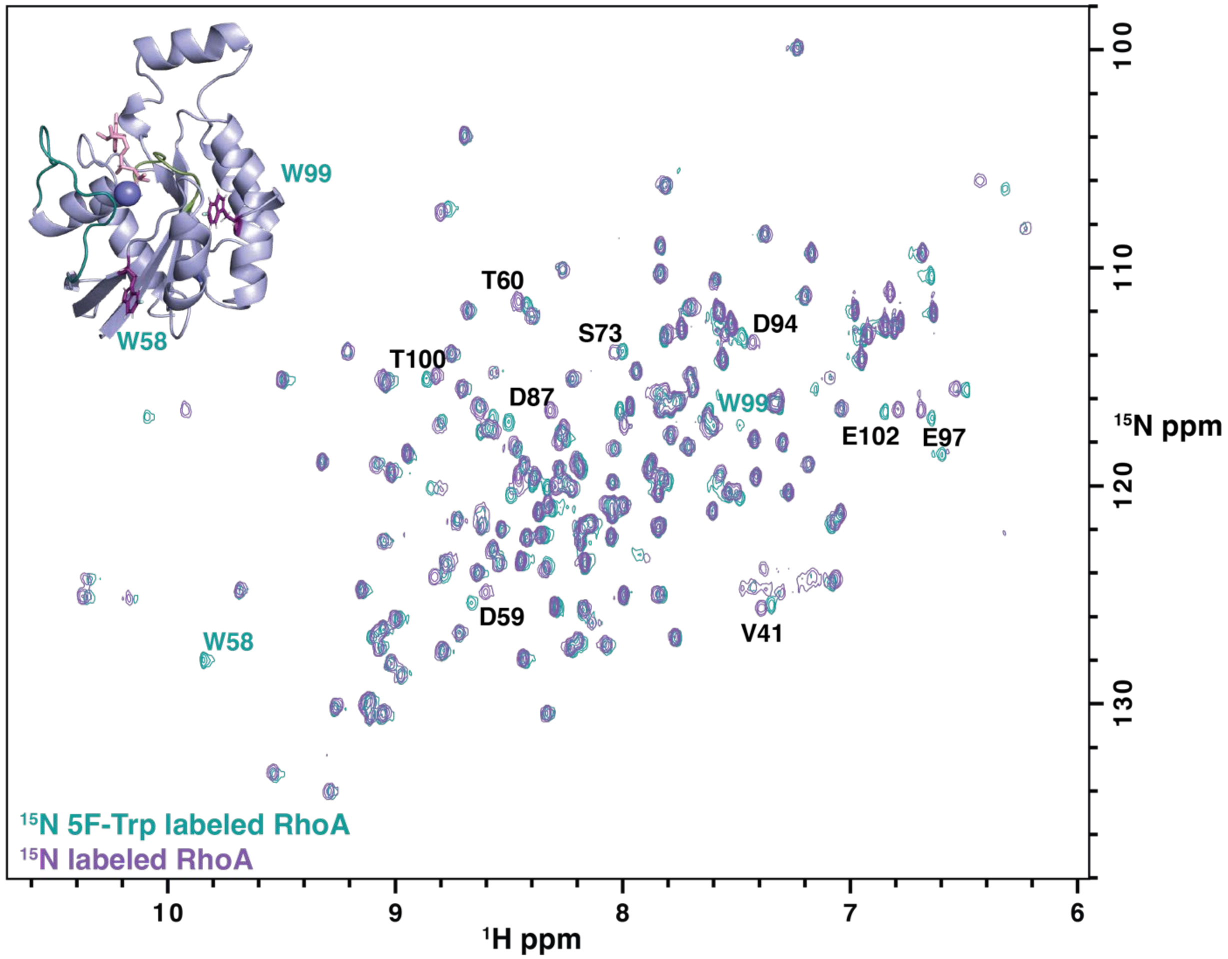

Figure S2: Superposition of 2D ^{1}H-^{15}N HSQC spectra of ^{15}N labeled RhoA (purple) and ^{15}N, 5F-Trp labeled RhoA (teal), with amide resonances of amino acids surrounding the two 5F-Trp residues W58 and W99 marked in black by residue name and number.

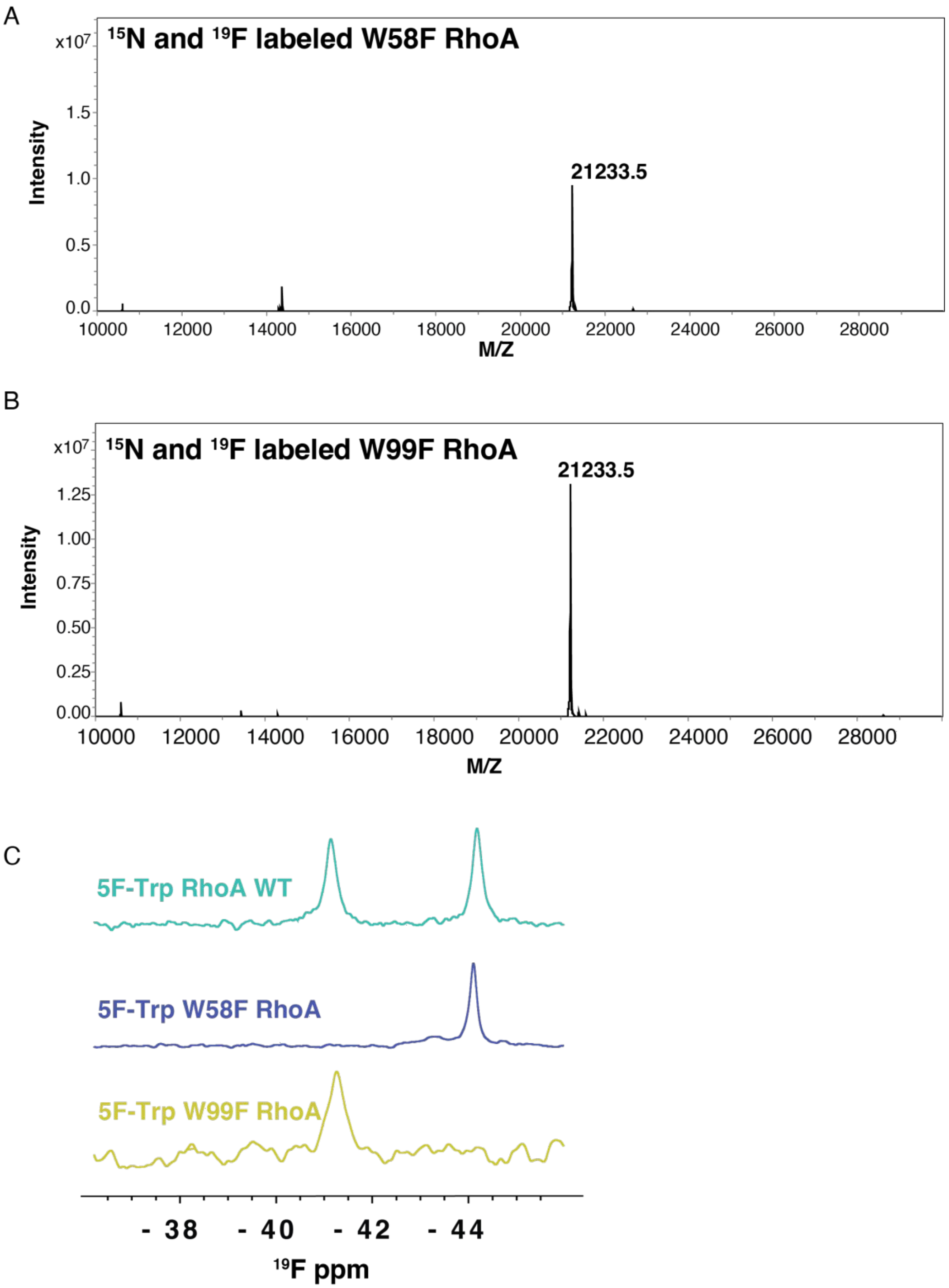

Figure S3: (A) ESI Mass spectrometry data of ^{15}N ^{19}F 5F-TrpW58F RhoA and (B) ^{15}N ^{19}F 5F-TrpW99F RhoA. The calculated expected mass for both is 21335 Da. (C) 1D ^{19}F spectra of 5F-Trp RhoA and W58F and W99F variants used for resonance assignment.

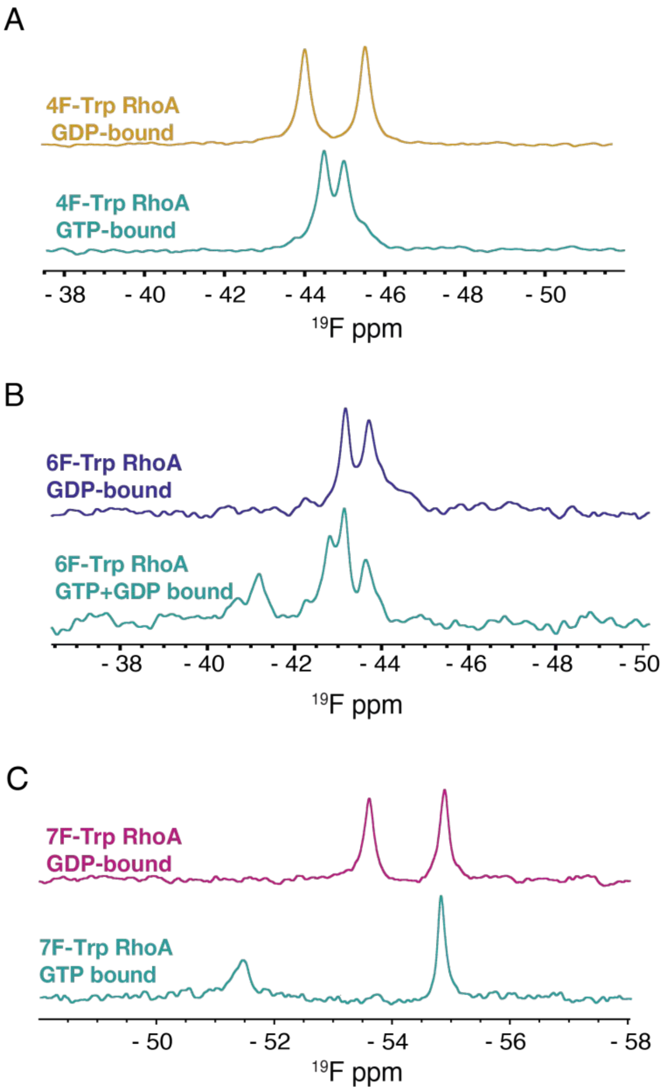

Figure S4: (A) 1D ^{19}F spectra of GDP-bound 4F-Trp RhoA protein (yellow) and GTP-bound 4F-Trp RhoA (green) with ^{19}F resonance frequencies of W58 and W99 at -44.95 ppm and -45.64 ppm, respectively, in the GDP-bound state and -44.40 ppm and -44.20 ppm, respectively, in the GTP-bound state. (B) 1D ^{19}F spectra of GDP-bound 6F-Trp RhoA (purple) and a mixture of GDP- and GTP-bound 6F-Trp RhoA (green) with ^{19}F resonance frequencies of W58 and W99 at -40.91 ppm and -41.45 ppm, respectively, in the GDP-bound state and -42.51 ppm and -41.95 ppm, respectively, in the GTP-bound state. (C) 1D ^{19}F spectra of GDP-bound 7F-Trp RhoA (magenta) and GTP-bound 7F-Trp RhoA (green) with ^{19}F resonance frequencies of W58 and W99 at -54.9 ppm and -53.88 ppm, respectively, in the GDP-bound state. The W99 resonance broadens and shifts downfield to -51.47 ppm in the GTP-bound state, while the W58 resonance is essentially unchanged.

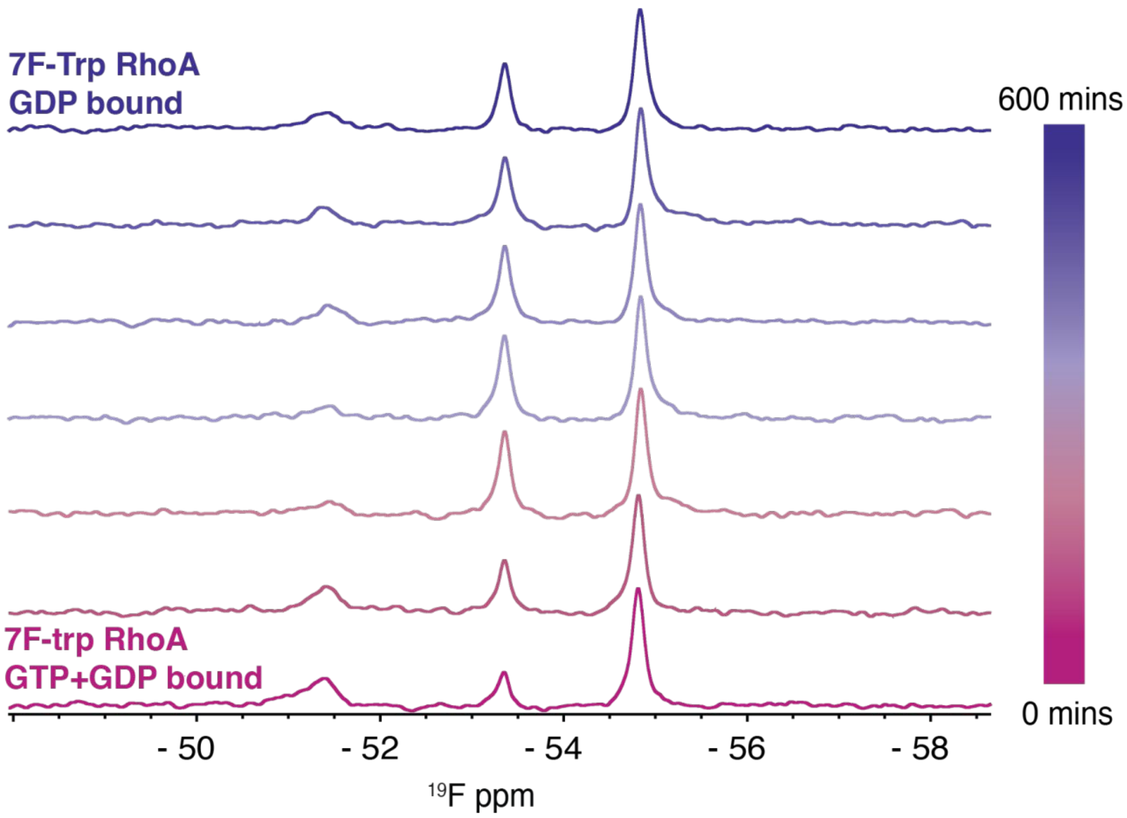

Figure S5: Time-dependent 1D ^{19}F spectra of 7F-Trp RhoA following GTP hydrolysis from 7F-Trp RhoA in the mixed GTP- and GDP-bound forms (pink) to 7F-Trp RhoA in the GDP-bound form (purple).

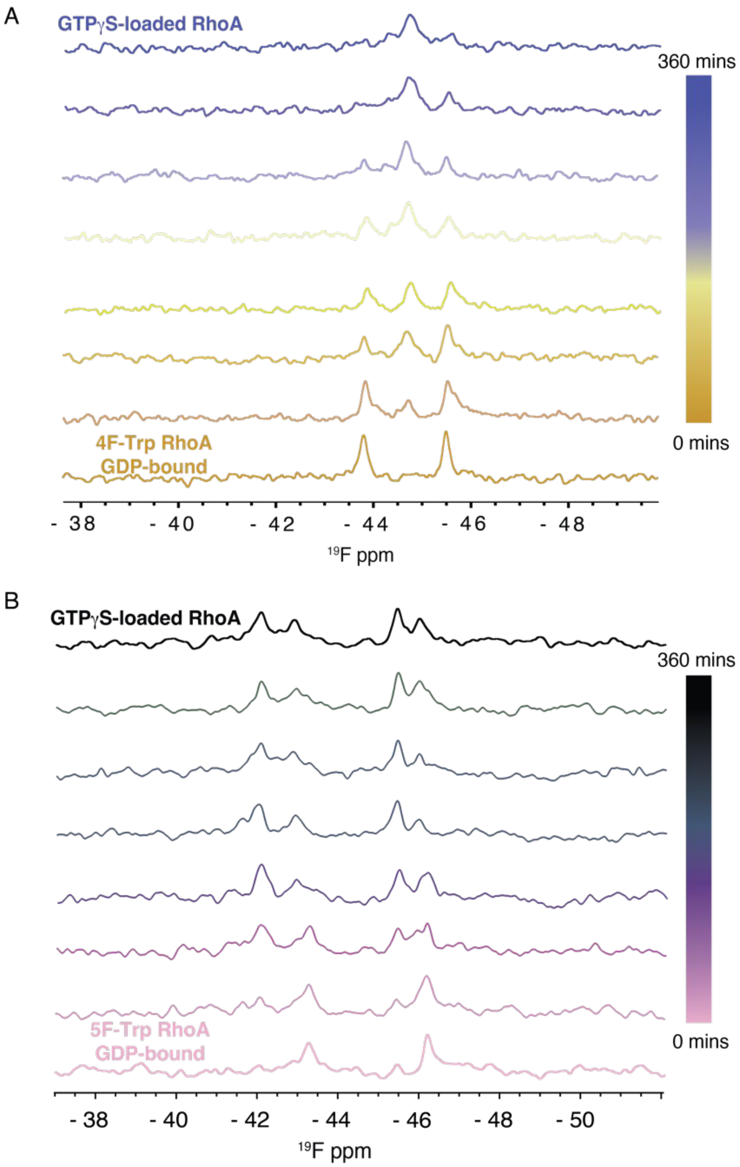

Figure S6: (A) Time-dependent series of 1D ^{19}F spectra of 4F-Trp RhoA-GDP undergoing nucleotide exchange from the GDP-bound form (yellow) to the GTPγS-bound form (blue) over 360 minutes. (B) Time-dependent series of 1D ^{19}F spectra of the 5F-Trp RhoA-GDP complex (pink) undergoing nucleotide exchange from GDP to GTPγS (black) over 360 minutes. Each sample was prepared by adding a 5-fold molar excess of GTPγS and 1D ^{19}F NMR spectra were recorded in five-minute increments.

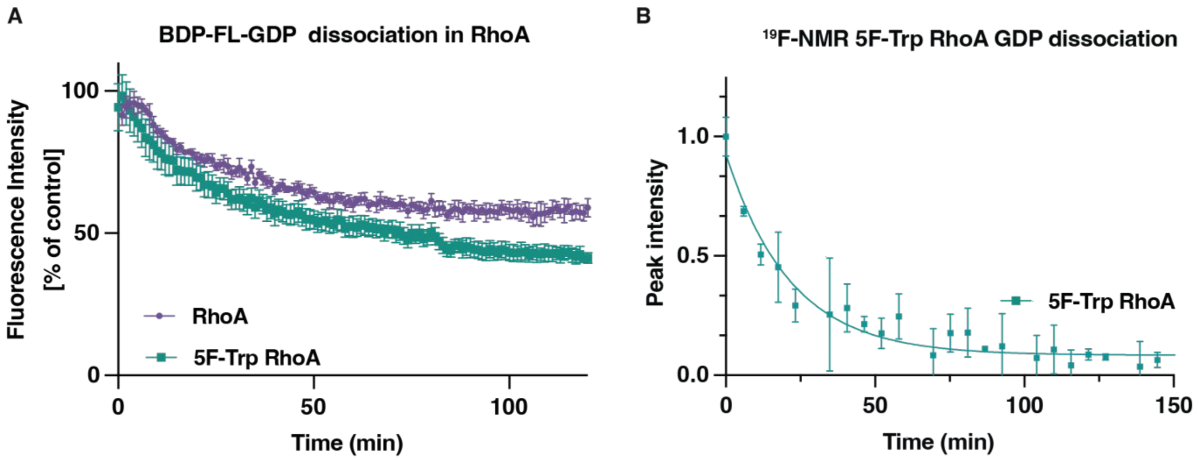

Figure S7: (A) Nucleotide dissociation assay for RhoA (purple) and 5F-TrpRhoA using a fluorescent GDP nucleotide. Data are plotted with error bars representing the mean ± standard deviation. The dissociation rate obtained for RhoA is 0.034 ± 0.004 min^{-1} and that for 5F-TrpRhoA is 0.029± 0.005. (B) Nucleotide exchange assay for exchange from GDP-to-GTPγS 5F-TrpRhoA (teal) using real-time ^{19}F spectra. The dissociation rate for 5F-TrpRhoA is 0.046 ± 0.007 min^{-1}. The solid line is the fit to a single exponential decay.

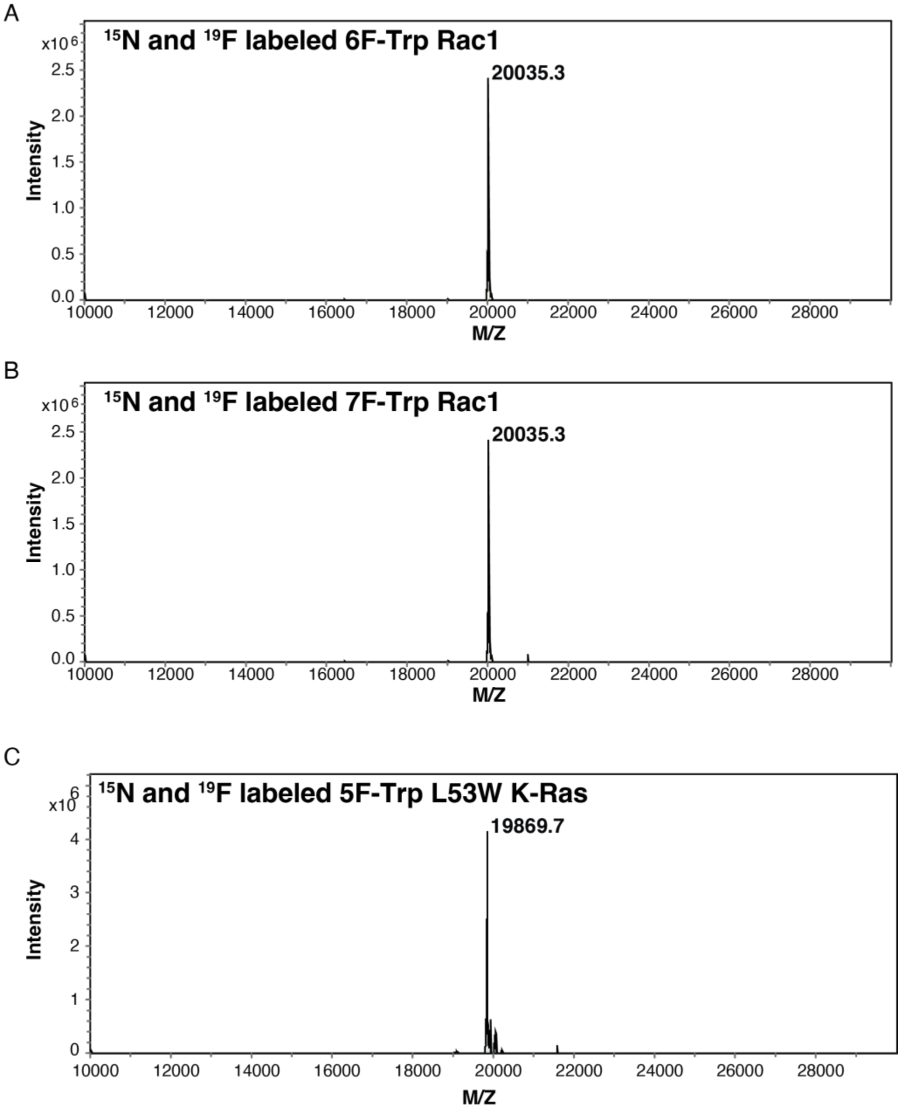

Figure S8: (A) ESI Mass spectrometry data of 6F-Trp ^{15}N ^{19}F labeled Rac1 and (B) 7F-Trp ^{15}N ^{19}F labeled Rac1 (the calculated expected mass for both is 20036 Da). (C) 5F-Trp ^{15}N ^{19}F labeled L53W K-Ras (the calculated expected mass is 19870 Da).

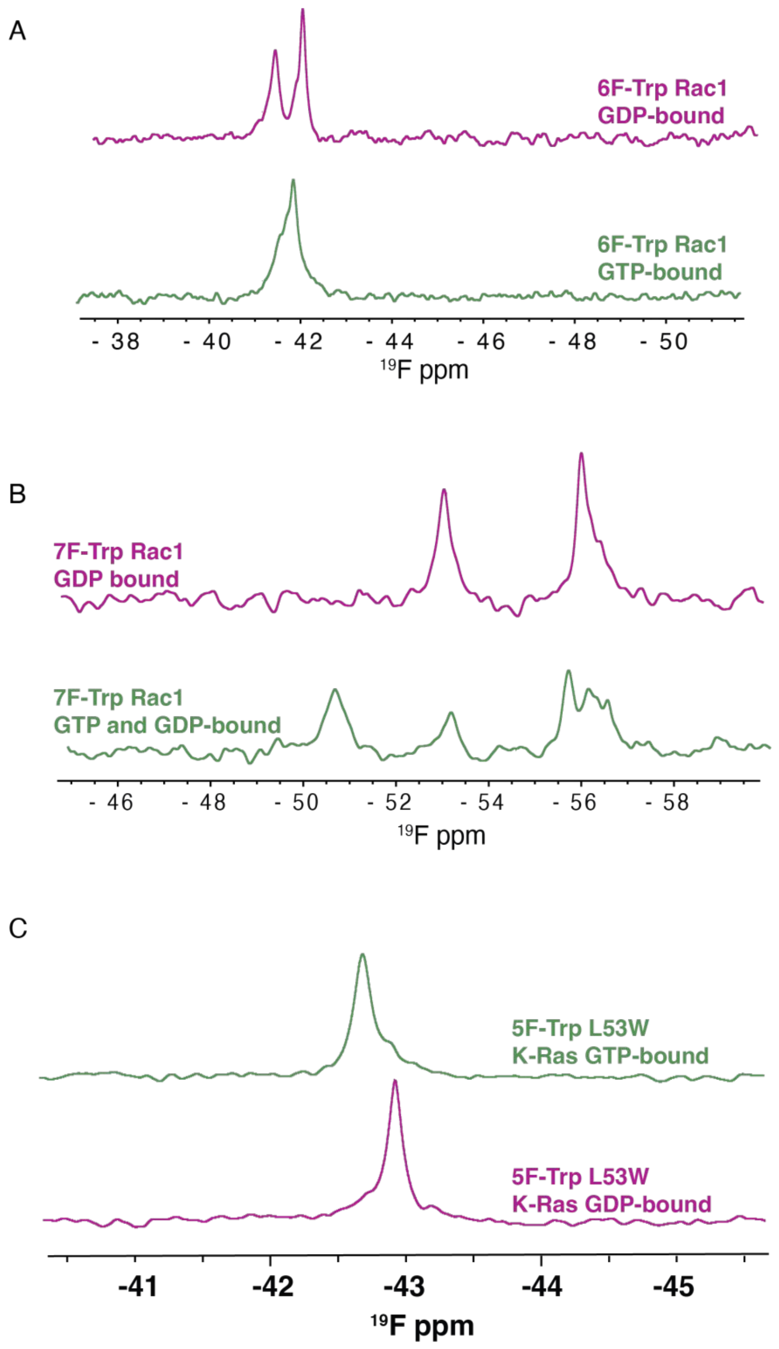

Figure S9 (A) 1D ^{19}F spectra of GDP-bound 6F-Trp Rac1 (magenta) and GTP bound 6F-Trp Rac1 (green). (B) 1D ^{19}F spectra of GDP-bound 7F-Trp Rac1 and GTP-bound 7F-Trp Rac1 (green). (C) 1D ^{19}F spectra of GDP 5F-Trp L53W K-Ras (magenta) and GTP-bound 5F-TrpL53W K-Ras (green).

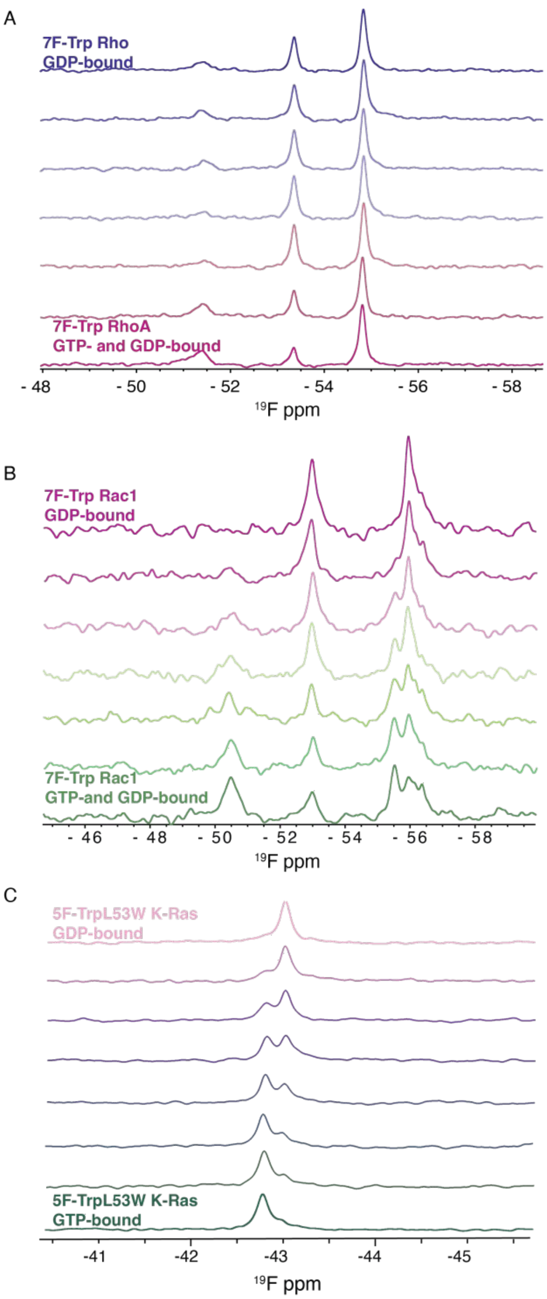

Figure S10 (A) Time-dependent series of 1D ^{19}F NMR spectra illustrating GTP hydrolysis in a mixture of GDP- and GTP-bound 7F-Trp RhoA (magenta). The final (top) spectrum is from the GDP-bound form (purple). (B) Time-dependent series of 1D ^{19}F NMR spectra illustrating GTP hydrolysis in a mixture of GDP-and GTP-bound 7F-Trp Rac1 (green). The final (top) spectrum is from the GDP-bound form (magenta) (C) Time-dependent series of 1D ^{19}F NMR spectra illustrating GTP hydrolysis from GTP-bound 5F-TrpL53W K-Ras (green) to GDP-bound (pink).

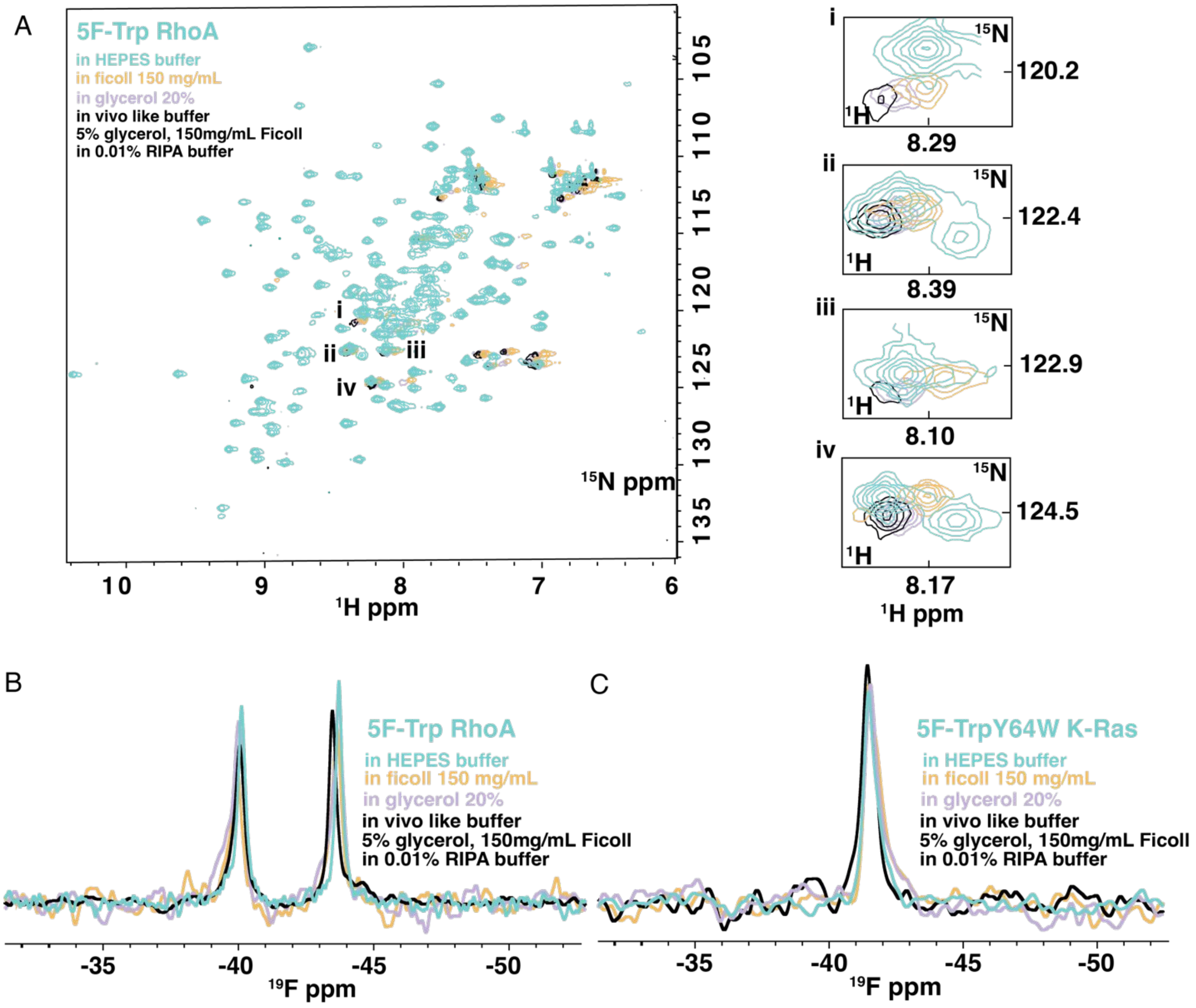

Figure S11 (A) 2D ^{1}H-^{15}N HSQC spectra of 5F-Trp RhoA under different buffer conditions and in the presence of several crowding agents: in HEPES buffer (teal), in the presence of 20% glycerol in HEPES buffer (purple), in the presence of 150 mg/mL Ficoll in HEPES buffer (yellow), and in buffer containing Ficoll 150 mg/mL, 5% glycerol and 0.01% RIPA buffer (black). (B, C) 1D ^{19}F spectra of 5F-Trp RhoA (B) and 5F-TrpY64W K-Ras (C) under the same conditions as in (A). No significant changes in the ^{19}F spectra occur in crowding conditions.

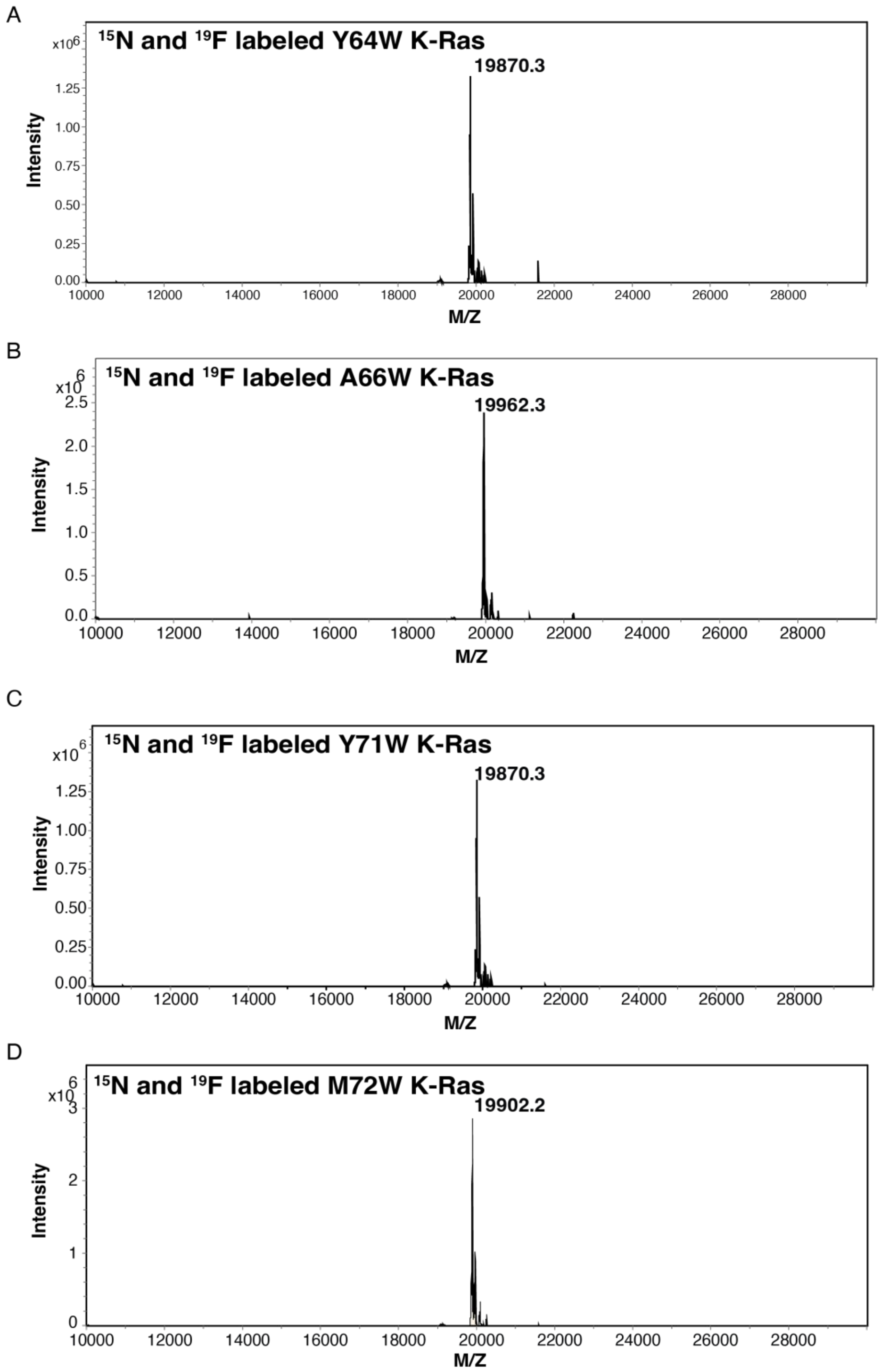

Figure S12: ESI Mass spectrometry data for four different variants of K-Ras (A) 5F-TrpY64W K-Ras, (B) 5F-TrpA66W K-Ras, (C) 5F-TrpY71W K-Ras, and (D) 5F-TrpM72W K-Ras. The calculated expected molecular masses are 19872 Da, 19964 Da, 19872 Da, and 19905 Da, respectively.

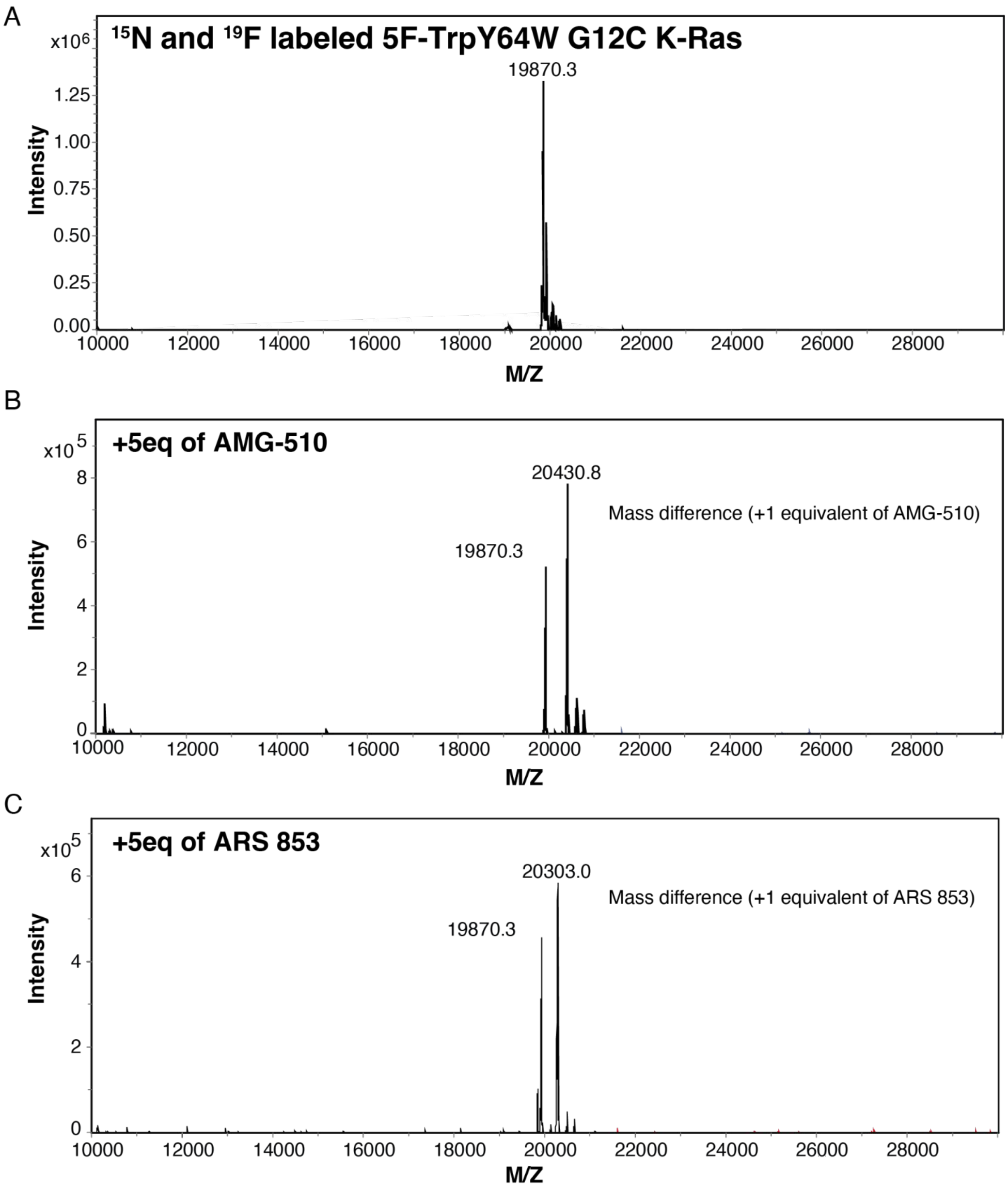

Figure S13: ESI Mass spectrometry data of the adduct of (A) 5F-TrpY64W G12C K-Ras with (B) AMG-510 or (C) ARS-853. The calculated expected molecular masses are 19871 Da, 20430 Da, and 20305 Da, respectively.

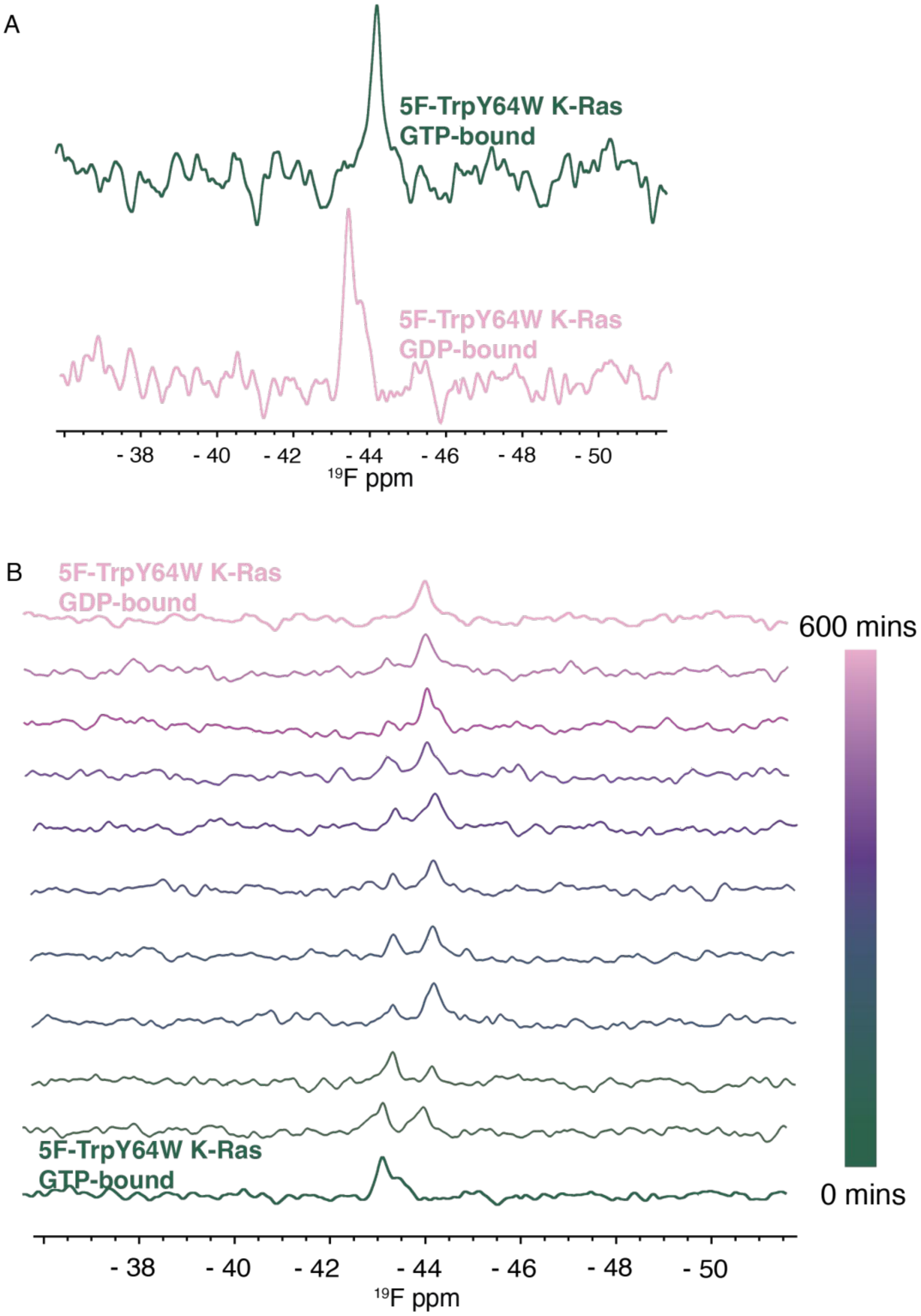

Figure S14: (A) 1D ^{19}F spectra of 5F-TrpY64W K-Ras bound to GDP (pink) and 5F-Trp labeled Y64W K-Ras bound to GTP (green). (B) Time dependent series of 1D ^{19}F NMR spectra illustrating GTP hydrolysis from GTP-bound 5F-TrpY64W K-Ras (green) to GDP-bound 5F-TrpY64W K-Ras (pink).

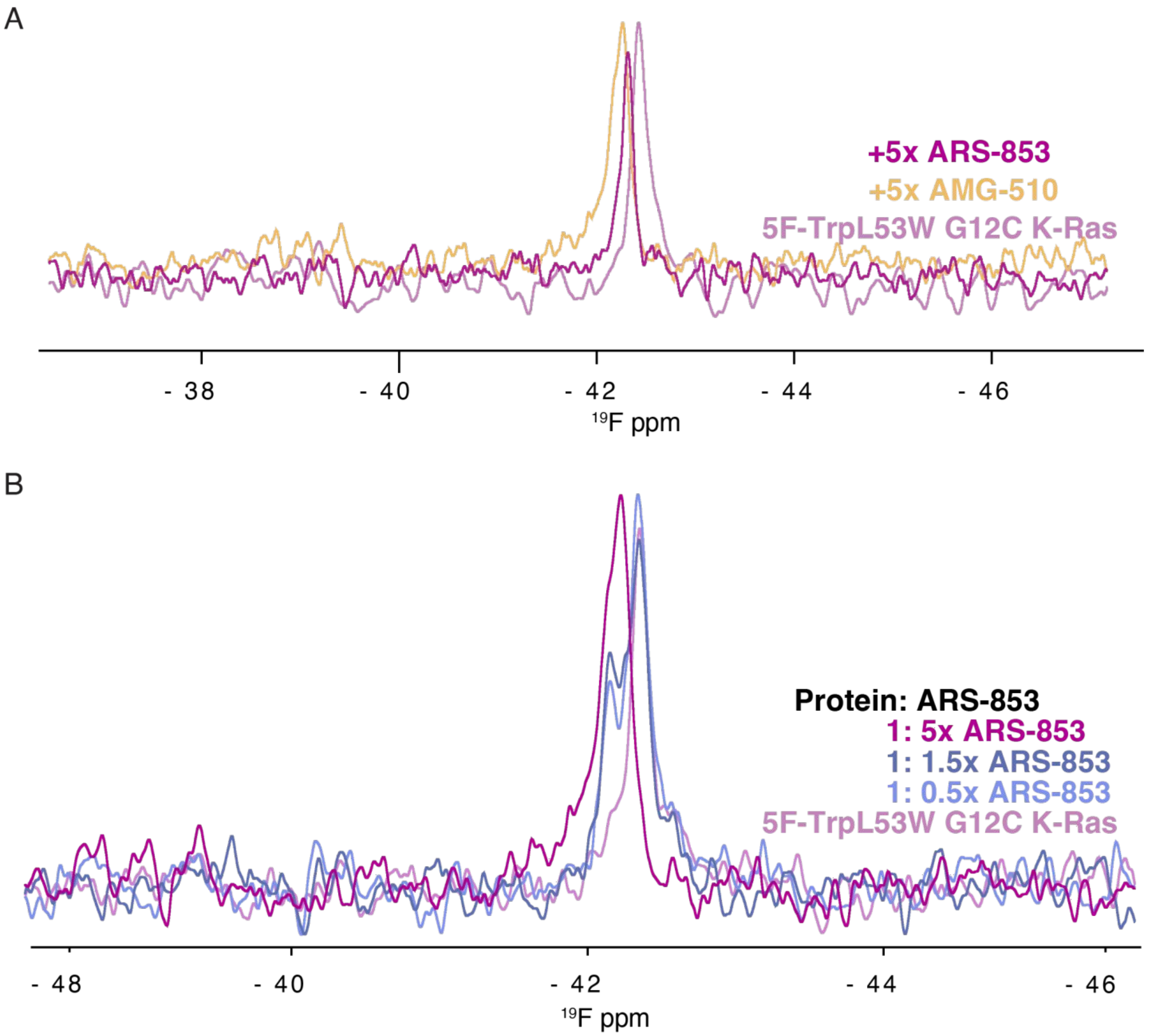

Figure S15: (A) 1D ^{19}F spectra of 5F-TrpL53W G12C K-Ras (pink) in the presence of AMG-510 (yellow) or ARS-853 (magenta). (B) 1D ^{19}F spectra of 5F-TrpL53W G12C K-Ras for three different ratios of ARS-853 to protein.

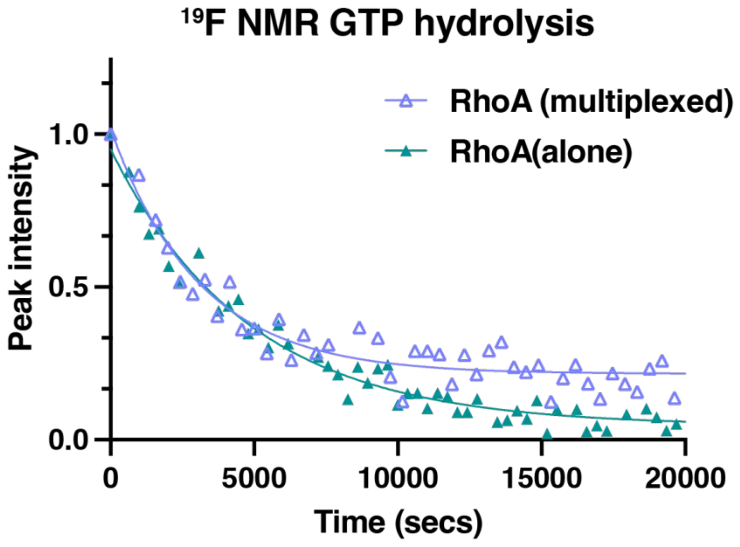

Figure S16: GTP hydrolysis by 6F-Trp RhoA alone (teal) compared to in the multiplexed assay (purple); the hydrolysis rate from these measurements is 0.019 min^{-1}

References

(1) Kelman, Z. *Isotope Labeling of Biomolecules–Labeling Methods*; Academic Press, 2015.
(2) Campos-Olivas, R.; Aziz, R.; Helms, G. L.; Evans, J. N.; Gronenborn, A. M. Placement of ^{19}F into the center of GB1: effects on structure and stability. *FEBS letters* **2002**, *517* (1-3), 55-60.
